# Navigating Towards a Well-Being Economy: Need for a Robust Theory of Change: Comment on "Can a Well-Being Economy Save Us?"

**DOI:** 10.34172/ijhpm.8873

**Published:** 2025-01-25

**Authors:** David G. Legge

**Affiliations:** School of Psychology and Public Health, La Trobe University, Melbourne, VIC, Australia.

**Keywords:** Well-Being Economics, Political Economy, NIEO, Theory of Change

## Abstract

In his recent editorial, Professor Labonté^1^ surveyed international initiatives calling for a well-being global economy. Most of these initiatives offer glowing visions but implausible theories of change. The 1974 United Nations call for a New International Economic Order (NIEO) provides a case study of an earlier instance of well-being economics (although not labelled as such). The NIEO included specific institutional and regulatory initiatives directed to achieving a fairer and more liveable world. However, it was defeated through the rise of neoliberalism from the 1980s as well as internal contradictions within the movement for a NIEO. The history of the NIEO provides useful lessons regarding the political dynamics of global transformation. Any policy initiatives, directed towards reforming the global economy for the well-being of people and planet, need to be based on a robust theory of change.

 Humanity and planet earth face multiple crises, including deepening inequality, pollution and biodiversity loss,^2^ and global warming.^3^

 In his recent editorial in this journal, Professor Labonté^1^ surveyed a number of initiatives directed to arresting the “business as usual” trajectory by building a well-being economy. His survey included two initiatives associated with World Health Organization (WHO) (the Council on the Economics of Health for All^4^ and the Global Framework for Well-being and Public Health^5^) as well as the Well-being Economy Governments Network (and the associated Well-being Alliance^6^), and the Earth for All initiative^7^ (sponsored by the Club of Rome).

 All of the initiatives surveyed acknowledge the polycrisis and recognise that the way in which the global economy works incorporates barriers to an effective and timely response. However, the policy prescriptions of these initiatives—how to get to a well-being economy—vary widely and are, in several cases, implausible.

 Navigating towards a well-being economy involves modelling political pathways to change, identifying the instabilities which open new possibilities, mapping the forces at play and tracing different scenarios of engagement. In short, a “theory of change.”^8^

 Labonté is too gentle in his review of these different initiatives in relation to their various “theories of change.” He concludes that:

 “*Plotting some escape routes out of capitalism is the biggest and most urgent challenge facing efforts to put well-being economics into substantive practice. Governments here will need the creativity and advocacy of a strong, mobilized civil society working alongside committed policy and political actors willing to confront powerful opponents.”*

 This is not the consensus across the various initiatives reviewed. In fact the theories of change espoused by most of these well-being initiatives surveyed are weak and do not offer any escape routes out of capitalism.

 The WHO Council relies heavily on exhortation and alternative metrics. Its final report includes 47 musts and 22 shoulds. The Council proposes that new metrics, to replace gross domestic product (GDP) as the lodestar of economic policy, will somehow drive the implementation of well-being policies. The Council’s premise is that “the economy” is presently governed on the basis of an inappropriate metric, GDP, which is a poor indicator of societal well-being. Hence the need to change the metric so that economic policy might be tied more closely to societal well-being.

 An alternative story would be that the economy is governed by the allocation of private capital which is guided by GDP as a predictor of profitability (of which it is a highly appropriate indicator). The role of GDP as a metric of particular interest to investors on account of its relationship to profit expectations is not considered.

 The 2015-2030 Sustainable Development Goals (SDGs) provide an earlier case of global change driven by improved metrics, collective exhortation, and rhetorical commitment. Unfortunately, the world is failing to make progress on most of the well-being indicators adopted for the SDGs.^9^ A lesson which could be drawn from the SDGs is the importance of rigorous analysis of the political dynamics of global change; in other words, a plausible theory of change.

 The Well-being Economy Alliance focuses on cultural change; it aims to bring together.

 “*Multiple actors across all regions, sectors and levels of our system to influence societal values and norms, and above all, to show that change is necessary and possible. Our underpinning value is that collaboration and togetherness define both our destination and also how we get there. Transformation calls for an entirely different way of being within human society: a shift from ‘us vs them’ to ‘WE All.’” *

 The limits of solidarity as a driver of global policy making were on show during the COVID-19 pandemic. Despite repeated calls by the Director General of WHO for solidarity in the face of the pandemic, the rich countries with Big Pharma and the big philanthropies put together a response, the Access to COVID-19 Tools (ACT) which led to the hoarding of vaccines by the rich countries and massive inequalities in access to vaccination.^10^

 The Well-being Economy Alliance and the WHO initiatives appear to assume that it has been the lack of policy directions that has held up progress rather than considering the processes and power relations of policy implementation and the structures of governance.

 Earth For All stands out among the initiatives reviewed, partly because of the clear account of contemporary neoliberal capitalism presented.^7^ The theory of change motivating Earth for All relies on social movements demanding that governments adopt new policies directed to societal well-being. This is a more plausible theory of change than moral exhortation and better metrics.

 One “well-being initiative” that Labonté could—perhaps should—have included in his review is the movement for a new New International Economic Order (NIEO) articulated in the 2014 Santa Cruz de la Sierra Declaration of the Group of 77 and China, For a new world order for living well.^11^

 The United Nations General Assembly adopted the original NIEO in 1974, in United Nations General Assembly Resolutions 3201 and 3202.^12^ The NIEO was sponsored by the Non-Aligned Movement and the G77.^13^ The NIEO was informed by dependency theory which argued that unfair trading relations were embedded in the structure of the global economy resulting in a net flow of value from South to North.^14^ Dependency theory suggested that developing countries should use tariffs and import quotas to make imported manufactures more expensive so that local producers could get established. It argued for subsidies and infrastructure supports to increase the competitiveness of exports from developing countries in world markets. Dependency theory also argued that the unequal exchange relations embedded in South North trade could be avoided through the cultivation of South South trade. The NIEO included a range of policy proposals regarding the governance of the global economy, based on dependency theory.

 The global regime of unequal exchange originated with colonialism. The net flow of value to the colonial powers contributed to capital accumulation in the metropolis and to the achievement of the high-income status that the erstwhile colonial powers enjoy today. While direct rule colonialism was rolled back through various struggles for national liberation the regime of unequal exchange was kept in place through the continuing power imbalance associated with economic imperialism.

 The promises of the NIEO did not survive the 1980s debt crisis, structural adjustment policies imposed by the International Monetary Fund in the context of debt bailouts, and by the provisions in various trade agreements under the World Trade Organization. The sweep of trade and investment agreements introduced since 1994 have progressively liberalised international trade and finance (although not the sharing of technical knowhow or the free movement of people).^15^

 The demands of the original NIEO reflected the priorities of governments in the global South, trying to achieve social and economic development in a regime of unequal exchange. It was also aligned with the interests of domestic capitalists seeking more equitable participation in global capitalism. This statist (and domestic capitalist) orientation was expressed in the contradictions between the progressive rhetoric of the NIEO advocates and the authoritarian character of many of their domestic regimes.

 The lack of any critique of extractivism or of the commitment of capitalism to unlimited economic growth, was also a weakness of the original NIEO. It also failed to directly address economic inequality within, as well as between, countries. While economic inequality reflects the workings of global capitalism, domestic capitalism plays a significant role in reproducing such inequality. Capitalism encourages ruling class elites to stoke the divisions associated with gender, ethnicity, class, disability and religion in order to divide, prevail and exploit. Imperialism contributes to the widening of economic inequality, due to the ways countries of the Global South are integrated in the global circuits of capital.

 A suite of policy proposals for the reform of global economic governance is currently being advanced by governments of the Global South, in particular through the G77 and in the discussions of the BRICS+. These demands, sometimes packaged as a new NIEO, include:

- special and differential treatment for low- and middle-income countries in the regulation of trade and global finance,
- a loss and damage funding mechanism to support adaptation to climate change,
- the regulation of transnational corporations,
- de-dollarisation and de-throning US control of international finance,
- equity and solidarity in pandemic preparedness and response (including loosening of the Agreement on Trade-Related Aspects of Intellectual Property Rights (TRIPS) disciplines, and making pathogen sharing conditional on benefit sharing),
- delinking the funding of pharmaceutical innovation from the prices charged for medicines.

However, the contradictions of the original NIEO have not disappeared. If the new NIEO is just about the capitalists of the Global South getting a fair go in the global economy, then it would be unlikely to do much about inequality, climate change, or societal well-being. On the other hand, if advocates of a new NIEO envisage moving beyond transnational capitalism, they can expect an even more desperate backlash from the imperium (Cuba and Venezuela offer stark warnings). Strong domestic support for the more radical agenda would be needed to proceed with the necessary structural reforms while withstanding the interventions of the empire.

 Labonté identifies the need for a strong, mobilized civil society working alongside committed policy and political actors to confront powerful opponents. This conclusion is brought into sharp focus in the light of these contradictions.

 Without the civil society mobilisation that Labonté calls for, the governments of the Global South will be limited to seeking more equitable participation in for domestic capital in global capitalism.

 The weak theories of change associated with most of the well-being initiatives reviewed by Labonté may be due to their avoidance of any modelling of the political dynamics of global change. Modelling the political dynamics of change would involve exploring possible scenarios of global change; identifying the systemic instabilities which could make space for progressive change; mapping the forces which take advantage of such windows of opportunity; and identifying the strategies which different stakeholders might adopt to strengthen the forces of progressive change. It is significant that none of the initiatives in review uses the term “imperialism.”

## Acknowledgements

 The author is a founding and active member of the People’s Health Movement (PHM). The discussion of the NIEO and some other sections in this paper build on text from the PHM paper, Confronting Capitalism and Imperialism (See Reference 15 in revised manuscript) of which I was the principal author (and the sole author of the sections which have been developed in the current submission).

## Ethical issues

 Not applicable.

## Conflicts of interest

 Author declares that he has no conflicts of interest.
